# Application of loop-mediated isothermal amplification for malaria diagnosis during a follow-up study in São Tomé

**DOI:** 10.1186/1475-2875-11-408

**Published:** 2012-12-06

**Authors:** Pei-Wen Lee, Dar-Der Ji, Chia-Tai Liu, Herodes S Rampao, Virgilio E do Rosario, I-Feng Lin, Men-Fang Shaio

**Affiliations:** 1 The Anti-Malaria Team of Taiwan in São Tomé and Príncipe, São Tomé and Princípe, Taipei, Taiwan; 2 Research and Diagnostic Center, Centers for Disease Control, Taipei, Taiwan; 3 Centro National de Endemias, São Tomé, Democratic Republic of São Tomé and Príncipe, Taipei, Taiwan; 4 Instituto de Higiene e Medicina Tropical/Universidade Nova de Lisboa, Lisbon, Portugal; 5 Institute of Public Health, National Yang-Ming University, Taipei, Taiwan; 6 Department of Tropical Medicine, School of Medicine, National Yang-Ming University, Taipei, Taiwan

**Keywords:** Malaria, Microscopy, Rapid diagnostic test, Nested polymerase chain reaction, Loop-mediated isothermal amplification, Treatment follow-up

## Abstract

**Background:**

A reliable and simple test for the detection of malaria parasite is crucial in providing effective treatment and therapeutic follow-up, especially in malaria elimination programmes. A comparison of four methods, including nested polymerase chain reaction (PCR) and loop-mediated isothermal amplification (LAMP) were used for the malaria diagnosis and treatment follow-up in São Tomé and Príncipe, during a successful pre-elimination campaign.

**Method:**

During the period September to November 2009, blood samples from 128 children (five to 14 years old) with temperature ≥38°C (tympanic) in the District of Agua Grande were examined using four different methods, i.e., histidine-rich protein 2 (HRP-2) based rapid diagnostic tests (HRP-2-RDTs), optical microscopy, nested PCR, and LAMP. First-line treatment with artesunate-amodiaquine was given for uncomplicated malaria and intravenous quinine was given for complicated malaria. Children with persistent positivity for malaria by microscopy, or either by nested PCR, or by LAMP on day 7 were given second-line treatment with artemether-lumefantrine. Treatment follow-up was made weekly, for up to four weeks.

**Results:**

On day 0, positive results for HRP-2-RDTs, microscopy, nested PCR, and LAMP, were 68(53%), 47(37%), 64(50%), and 65(51%), respectively. When nested PCR was used as a reference standard, only LAMP was comparable; both HRP-2-RDTs and microscopy had moderate sensitivity; HRP-2-RDTs had poor positive predictive value (PPV) and a moderate negative predictive value (NPV) for the treatment follow-up. Seventy-one children with uncomplicated malaria and eight children with complicated falciparum malaria were diagnosed based on at least one positive result from the four tests as well as clinical criteria. Twelve of the 79 children receiving first-line treatment had positive results by nested PCR on day 7 (nested PCR-corrected day 7 cure rate was 85%). After the second-line treatment, nested PCR/LAMP-corrected day 28 cure rate was 83% for these 12 children.

**Conclusions:**

HRP-2-RDTs have similar sensitivity as microscopy but less specificity. However, as compared to nested PCR, the poor sensitivity of HRP-2-RDTs indicates that low parasitaemia may not be detected after treatment, as well as the low specificity of HRP-2-RDTs indicates it cannot be applied for treatment follow-up. LAMP has similar sensitivity and specificity to nested PCR. With high PPV and NPV, LAMP is simpler and faster as compared to nested PCR with the advantage of detecting low parasitaemia becoming a potential point-of-care test for treatment follow-up.

## Background

Global control efforts have resulted in a reduction in the incidence of malaria and malarias specific mortality rates. Transmission of malaria decreased remarkably in the Republic of São Tomé and Príncipe (STP) since an integrated malaria control programme was scaled up beginning in 2004 [1,2]. While the decline in malaria morbidity and mortality is notable, potential threat of malaria epidemics in the status of a low transmission is of major concern because most of the asymptomatic carriers are not treated [3]. Asymptomatic carriers not only act as reservoirs for malaria transmission but also act as a risk factor for symptomatic attacks [4-8]. A prerequisite in the attempt to eliminate malaria from an endemic area is the identification of asymptomatic infections for treatment [9,10]. Therefore, provision of accurate diagnosis as well as prompt and effective treatment is the core of malaria elimination strategies in the country.

In April 2008, a cross-sectional nationwide malaria surveillance in STP by use of histidine-rich protein 2 (HRP-2) based rapid diagnostic tests (HRP-2-RDTs) showed a positive rate of 3.5%, which was further confirmed by microscopy [3]. Ninety percent of these malaria positive cases are asymptomatic and only half of them were cured with artemisinin-combination therapy (ACT) as confirmed with a follow up study. Follow-up results were verified by polymerase chain reaction (PCR) if inconsistent findings were observed between RDT and blood films. Failure treatment of the asymptomatic malaria carriers attributed to the non-compliance for treatment. This might be responsible for the increase in malaria cases observed in São Tomé in 2009, which was reversed after intensification of control measures [3].

The diagnostic testing effort in STP is high. The annual blood examination rate exceeds 30% on average, far greater than in other countries in the region [2]. However, current surveillance systems rely on diagnosis by microscopy or use of RDTs that are not sufficiently specific or sensitive to detect low-parasite-density infections. Microscopy and RDTs both become relatively insensitive at parasite densities below 100 parasites/μl [11-13]. In addition to the low sensitivity, the high false positive rate of HRP-2-RDTs is the major limitation to apply for monitoring of the therapeutic response [14]. To illustrate the importance of the statistical analysis in the evaluation of the accuracy of malaria diagnostic tests, Bayesian Latent Class Models were used to estimate the malaria infection prevalence, together with sensitivities, specificities, and predictive values of three diagnostic tests (HRP-2-RDTs, microscopy and nested PCR) and found that not only microscopy has poor sensitivity compared to the other tests but also caution should be taken with the positive predictive values (PPV) of the HRP-2-RDTs [15].

PCR-based molecular methods are good for both sensitivity and specificity but too sophisticated and expensive to be applied in most malaria-endemic countries [16]. The recently developed loop-mediated isothermal amplification (LAMP) method is cheaper, simpler, and faster. The LAMP reaction can be conducted under isothermal conditions and is almost as specific and sensitive as the nested PCR method for Plasmodium-DNA detection in blood [17-19]. In this study, the role of LAMP in the malaria diagnosis and therapeutic follow-up for children with ACT treatment was elucidated by comparing four diagnostic methods.

## Methods

### Patients and setting

In STP, an integrated malaria control programme was officially initiated in 2004, and a molecular diagnostic laboratory was set in the main island of São Tomé in 2007, following STP Government directives for malaria control and for ethical clearance throughout the implementation of the programme. Ethical approval was obtained from the Ministry of Health of the Democratic Republic of STP. Informed verbal consent was obtained from parents/guardians. Most parents/guardians are illiterate or semi-literate, therefore could not sign a written consent. This study took into account that the principles of verbal informed consent were the same for written informed consent. The work was approved by the Ministry of Health of STP and coordinated by the Centro Nacional de Endemias (CNE).

During the period September to November 2009, of 191 school children (five to 14 years old) from the district of Agua Grande presented to the health stations with temperature ≥38°C (tympanic), 128 completed four malaria diagnostic tests (HRP-2-RDTs, microscopy, nested PCR, and LAMP) and treatment follow-up. The other 63 febrile children (33%) without completing the four malaria tests were examined using only HRP-2-RDTs and/or optical microscopy, but were not included in this study. Failure to obtain blood spots on the filter paper from these children for nested PCR and LAMP was due to the refusal by their parents/guardians for sample collection.

### Blood collection

A fingerprick blood sample was obtained for the HRP-2-RDTs (ICT Malaria-Combo, batch 32185, ICT Diagnostics, South Africa) in addition to thick and thin films. An additional 3–5 drops (~125 μl) of blood were spotted onto filter paper (FTA, classic card, Whatman), which were collected and sent to the molecular diagnostic laboratory for subsequent nested PCR and LAMP analysis.

### Microscopy

Thick and thin films were stained with 10% Giemsa for 10 min and microscopic reading on blood films was performed according to CNE diagnostic protocols. Two trained microscopists examined the blood films simultaneously and a third one clarified any discrepancy in results. A blood film was declared negative when no parasite was detected in 200 fields. Estimation of parasitaemia was made by count of 500 leucocytes and then expressed as the number of asexual parasites per microlitre by assuming a leukocyte count of 8,000/μl. The microscopists were blinded to any clinical diagnosis, results of HRP-2-RDTs, nested PCR, and LAMP during the course of this study.

### HRP-2-RDTs

HRP-2-RDTs were prepared and read by nurses and then read by technicians and/or physicians. All readers were trained to perform the tests according to manufacturer’s instructions. Physicians interpreted and recorded RDT results as either positive or negative after 15 min; they were trained to consider faint test lines as positive.

### Nested PCR assay

The dry blood spot in a circle size with one inch diameter (~ 125 μl whole blood) on the filter paper was excised and cut into four quarters, one quarter for nested PCR and one quarter for the LAMP. For the nested PCR, DNA was extracted from the quarter dry blood spot using the QIAamp DNA Blood Mini Kit (Qiagen Inc., Valencia, CA, USA) according to manufacturer’s protocol. The DNA was finally eluted with 50 μl distilled water. Nested PCR amplification was performed as described previously by Snounou [20]. In brief, detection and speciation of Plasmodium is done with a two-step nested PCR. In the first step, 2.5 μl of extracted DNA is amplified using genus specific primers; in the second step, 1 μl of the first PCR amplification product is further amplified using primers specific for each Plasmodium species. Ten microlitres of each second PCR amplified DNA product is electrophoretically resolved on a 2% agarose gel, stained for 15 min with ethidium bromide and visualized by UV illumination for analysis of results.

### LAMP assay

For the LAMP, a rapid DNA extraction method for dry blood spot was modified from the method previously described by Bereczky *et al.*[21]. In brief, the quarter dry blood spot was placed into a 1.5 ml centrifuge tube and cut into small pieces with a pair of scissors. The filter pieces were washed twice with 0.5 ml of FTA® Purification buffer (Life Technologies, Gaithersburg, MD, USA) for 5 min, twice with 0.5 ml of 10 mM Tris (pH 8.0) containing 0.1 mM EDTA for 5 min and again air-dried on a 56°C heating block. Some 100 μl of double distilled water added, left to stand for 5 min, and then heated for 15 min at 100°C. The supernatant, containing DNA, was subsequently used in visualized LAMP.

One LAMP primer sets for *Plasmodium* genus and four species specific LAMP primer sets for each of four human malaria species reported by Han *et al.* were used in this study [18]. The duplicate reaction mixtures (25 μl) contained 5 μl of the extracted DNA, 1 μl of Bst DNA polymerase, and 1 μl of fluorescent detection reagent (Eiken Chemical Co., Ltd., Tokyo, Japan) in 1 × reaction buffer [20 mM Tris–HCl (pH 8.8), 10 mM KCl, 8 mM MgSO_4_, 10 mM (NH4)_2_SO_4_, 0.1% Tween 20, 0.8 M Betaine, a 1.4 mM concentration of each dNTP] and the inner primers FIP and BIP at 1.6 μM, the loop primers LPF and LPB at 0.8 μM and the outer primers F3 and B3 at 0.2 μM. Amplification was performed at 60°C with a heat block (Heatblock III, VWR International, Chicago, IL, USA) for 100 min and then heated at 90°C with a heat block for 1 min to terminate the reaction. Duplicate negative control (water) and positive control (water spiked with DNA) were included in each run.

The positive LAMP reaction was detected by observing the fluorescence in the reaction tube via naked eye under a portable UV lamp [22]. In this study, an interval of 10 min was set to determine the threshold time of the LAMP reaction. The threshold time for positivity was found at 60 min and the optimal reaction time was determined at 100 min, as positive samples with low parasitaemia showed positivity within 100 min. False positivity was usually seen when the reaction time was over 120 min. Two technicians conducting nested PCR and LAMP independently were blinded to any clinical diagnosis or results from HRP-2-RDTs/microscopy. Both nested PCR and LAMP were performed in duplicate. If the results were inconsistent in each duplicate reaction, a third run was carried out to confirm the results.

### Treatment and follow-up

All patients were seen and the assays, except DNA amplification, were performed on site at the health stations. Blood spots on the filter paper for nested PCR and LAMP were transferred to the molecular diagnostic laboratory within 1 hr after collection. Blood samples for nested PCR and LAMP were treated and assayed individually on the collection day rather than batched and performed at a later time. Time to obtain test results varied depending on the different methods, i.e. 20 min for HRP-2-RDTs, 60 min for microscopy, 3 hr for LAMP, and 8 hr for nested PCR. Diagnosis of malaria was made as a febrile child had at least one positive result of the four malaria tests. Treatment was started as soon as possible at the time when the test results were available. Children with uncomplicated malaria were treated with a three-day course of artesunate-amodiaquine at home except for the first dose taken in front of the nurse’s observation. Children with severe malaria were admitted to hospital and treated with quinine intravenously. Treatment was switched to oral administration as soon as the patient was able to tolerate it. An additional course of primaquine was given for vivax malaria. Home treatment was weekly followed up by a mobile team (consisting of a nurse and a technician), which actively visited patients by taking blood samples for HRP-2-RDTs, optical microscopy, nested PCR and LAMP. Treatment failure was considered if parasitaemia persisted (microscopy positive), or either the nested PCR or the LAMP was positive. When treatment failures occurred after one week of initial treatment, artemether-lumefantrine (Coartem®, Novartis, Swiss) was given, with a three-week follow-up.

### Statistical analysis

The sensitivity and specificity of the four methods were calculated using either microscopy or nested PCR assay as a reference test. The percentage specificity, sensitivity, PPV, and negative predictive value (NPV) were calculated as reported previously [23]. Ninety-five percent confidence intervals (95%CI) for sensitivity, specificity, PPV, and NPV were calculated by an exact method for binomial distribution using SAS statistical package version 9.2 (SAS Institute Inc., Cary, NC, USA). In addition, the degree of agreement between two diagnostic tests was measured by the concordance response rate (percentage of responses with both positive or both negative results) and a Kappa statistic, which was ranged from 0 if the two tests were completely independent and to 1 if the two tests had perfect agreement.

## Results

Of the 128 febrile children where a complete set of four malaria tests were carried out, positive results for HRP-2-RDTs, microscopy, nested PCR, and LAMP, were 68(53%), 47(37%), 64(50%), and 65(51%), respectively (Table 1). Among the 68 positives for HRP-2-RDTs, there were 23 (34%) negatives for microscopy which were either positive for nested PCR and LAMP (10 children) or negative for nested PCR and LAMP (13 children). On the other hand, two were HRP-2-RDT negative but positive for microscopy, nested PCR, and LAMP. Among the 45 positives for both HRP-2-RDTs and microscopy, one was negative for both nested PCR and LAMP. Of the 23 negatives for microscopy but positives for HRP-2-RDTs, 10 were positive for both nested PCR and LAMP. Fourteen children with negative nested PCR were positive for falciparum malaria by HRP-2-RDTs (Table 1).

**Table 1:** Results of four malaria tests for febrile children before and after treatment

**HRP-2-RDTs**	**Microscopy**	**Nested PCR**	**LAMP**
**Before treatment**			
positive: 68	positive: 45	positive: 44	positive: 44
		negative: 1	negative: 1
	negative: 23	positive: 10	positive: 10
		negative: 13	negative: 13
negative: 60	positive: 2	positive: 2	positive: 2
		negative: 0	negative: 0
	negative: 58	positive: 8	positive: 8
		negative: 50	positive: 1
			negative: 49
**On day 7 after first-line treatment**			
positive: 33	positive: 6	positive: 6	positive: 6
		negative: 0	negative: 0
	negative: 27	positive: 1	positive: 1
		negative: 26	negative: 26
negative: 46	positive: 1	positive: 1	positive: 1
		negative: 0	negative: 0
	negative: 45	positive: 4	positive: 3
			negative: 1
		negative: 41	negative: 41

Among the 58 children with negative results for both HRP-2-RDTs and microscopy, eight were positive for both nested PCR and LAMP, but one with negative nested PCR was positive for LAMP (the only one single infection with *Plasmodium malariae* found in this study). Species identification based on nested PCR and LAMP showed that 62 children exhibited single infection with *Plasmodium falciparum* and two children were mixed infections (one with *P. falciparum* and *P. malariae*, the other with *P. falciparum* and *Plasmodium vivax*)*.* Optical microscopy showed that the parasite density ranged widely, 100–100,000/ul for 47 children on day 0, with a narrower range of 100–10,000/ul in seven children on day 7.

Malaria positive cases were defined from one positive result out of the four tests. Taking together, 79 children were positive for malaria; 71 uncomplicated and eight complicated. The initial treatment was followed up on day 7, numbers of the positive results for HRP-2-RDT, microscopy, nested PCR, and LAMP, were 33, seven, 12, and 11, respectively (Table 1). Of the 79 children receiving treatment, 67 children with negative microscopy were also negative for nested PCR and LAMP, which was considered as effective treatment (a cure rate of 91% assayed by microscopy but 85% corrected by nested PCR and 86% by LAMP). Further weekly follow-up for these children was discontinued due to the refusal by most of the children’s parents for sample collections. On the other hand, 12 children found positive for nested PCR on day 7 after the initial treatment were regarded as treatment failure and received a second-line treatment with artemether-lumefantrine (Table 2). Children A and K did not complete the first-line treatment because their parents/guardians considered that the children had improved their health conditions. On day 14 (one week after the second-line treatment), cure rate for these 12 children reached 100% if based upon microscopy but 92% if corrected by nested PCR and LAMP (Table 2). On day 28, cure rate showed 100% if determined by microscopy but 83% if corrected by nested PCR and LAMP. Child A remained malaria positive constantly by nested PCR and LAMP throughout the four-week follow-up, despite a good response to the second-line treatment as judged by microscopy. This indicates the possible existence of falciparum parasites with lower susceptibility to the drug treatment. Child K who did not take the full course of first-line treatment was negative by microscopy but positive by nested PCR and LAMP on day 7, had a temporary response to the second-line treatment on day 14 when evaluated by nested PCR and LAMP, suggesting again presence of falciparum parasites that do not respond to the treatment, demanding a more detailed study in drug resistance in the area.

**Table 2:** Results of four malaria tests for patients receiving second-line malaria treatment on day 7 with weekly follow-up

**Case**	**HRP-2-RDTs**				**Microscopy**				**Nested PCR**				**LAMP**			
	7	14	21	28	7	14	21	28	7	14	21	28	7	14	21	28
A	+	+	-	-	+	-	-	-	+	+	+	+	+	+	+	+
B	+	+	+	+	+	-	-	-	+	-	-	-	+	-	-	-
C	+	+	+	+	+	-	-	-	+	-	nd	-	+	-	nd	-
D	+	+	+	-	+	-	-	-	+	-	-	-	+	-	-	-
E	+	+	-	-	+	-	-	-	+	-	-	-	+	-	-	-
F	+	+	-	-	+	-	-	-	+	-	-	-	+	-	-	-
G	+	+	+	+	-	-	-	-	+	-	-	-	+	-	-	-
H	-	-	-	-	+	-	-	-	+	-	-	-	+	-	-	-
I	-	-	-	-	-	-	-	-	+	-	-	-	+	-	-	-
J	-	-	+	+	-	-	-	-	+	-	-	-	+	-	-	-
K	-	-	-	+	-	-	-	-	+	-	+	+	+	-	+	+
L	-	-	nd	-	-	-	nd	-	+	-	nd	-	-	-	nd	-

Case I was single infection with *P. malariae*; case L was mixed infection with *P. falciparum* and *P. vivax*; other cases were monoinfection with *P. falciparum*.

+: positive; -: negative.

nd: not done.

To elucidate the accuracy of the four tests for malaria diagnosis, either microscopy or nested PCR is used as a gold standard and sensitivity, specificity, PPV, and NPV are the indicators for comparison. When microscopy is used as a gold standard, both specificity and PPV were moderate for HRP-2-RDTs, nested PCR, and LAMP (Table 3). When nested PCR is used as a gold standard, only LAMP is comparable (Kappa = 0.98, Table 4); HRP-2-RDTs revealed moderate sensitivity (84%, 95%CI: 75.3-90.6%), specificity (78%, 95%CI: 68.6-85.7%), PPV (79%, 95%CI: 69.7-86.5%), and NPV (83%, 95%CI: 74.2-89.8%). Microscopy also had moderate sensitivity (72%, 95%CI: 62.1-80.5%) and NPV (78%, 95%CI: 68.6-85.7%).

**Table 3:** Agreement, sensitivity, specificity, PPV, and NPV of various tests for malaria diagnosis versus gold standard microscopy

**Tests**	**% Agreement with microscopy(Kappa)**	**Sensitivity**	**Specificity**	**PPV**	**NPV**
		**(95% CI)**	**(95% CI)**	**(95% CI)**	**(95% CI)**
**Before treatment**					
HRP-2-RDTs	80%	96	72	66	97
	(0.62)	(90.1, 98.9)	(62.1, 80.5)	(55.9, 75.2)	(91.5, 99.4)
Nested PCR	85%	98	78	72	98
	(0.70)	(93.0, 99.8)	(68.8, 85.7)	(62.1, 80.5)	(93.0, 99.8)
LAMP	84%	98	77	71	98
	(0.69)	(93.0, 99.8)	(67.5, 84.8)	(61.1, 79.6)	(93.0, 99.8)
**On day 7 after first-line treatment**					
HRP-2-RDTs	65%	86	63	18	98
	(0.18)	(77.6, 92.1)	(52.8, 72.4)	(11.0, 27.0)	(93.0, 99.8)
Nested PCR	94%	100	93	58	100
	(0.70)	(96.4, 100)	(86.1, 97.1)	(47.7, 67.8)	(96.4, 100)
LAMP	95%	100	94	64	100
	(0.75)	(96.4, 100)	(87.4, 97.8)	(53.8, 73.4)	(96.4, 100)

Percentage of agreement was measured by the percentage of test results with both positive or with both negative responses and its corresponding Kappa statistic (the higher the better; see texts in statistical analysis).

*HRP-2-RDTs*, histidine-rich protein 2 based rapid diagnostic tests; *Nested PCR*, nested polymerase chain reaction; *LAMP*, loop-mediated isothermal amplification; *PPV*, positive predictive value; *NPV*, negative predictive value.

**Table 4:** Agreement, sensitivity, specificity, PPV, and NPV of various tests for malaria diagnosis versus gold standard nested PCR

**Tests**	**% Agreement with nested PCR (Kappa)**	**Sensitivity**	**Specificity**	**PPV**	**NPV**
		**(95% CI)**	**(95% CI)**	**(95% CI)**	**(95% CI)**
**Before treatment**					
HRP-2-RDTs	81%	84	78	79	83
	(0.63)	(75.3, 90.6)	(68.6, 85.7)	(69.7, 86.5)	(74.2, 89.8)
Microscopy	85%	72	98	98	78
	(0.70)	(62.1, 80.5)	(93.0, 99.8)	(93.0, 99.8)	(68.6, 85.7)
LAMP	99%	100	98	98	100
	(0.98)	(96.4, 100)	(93.0, 99.8)	(93.0, 99.8)	(96.4, 100)
**On day 7 after first-line treatment**					
HRP-2-RDTs	61%	58	61	21	89
	(0.11)	(47.7, 67.8)	(50.7, 70.6)	(13.5, 30.3)	(81.2, 94.4)
Microscopy	94%	58	100	100	93
	(0.70)	(47.7, 67.8)	(96.4, 100)	(96.4, 100)	(86.1, 97.1)
LAMP	99%	92	100	100	99
	(0.95)	(84.8, 96.5)	(96.4, 100)	(96.4, 100)	(94.6, 99.9)

Percentage of agreement was measured by the percentage of test results with both positive or with both negative responses and its corresponding Kappa statistic (the higher the better; see texts in statistical analysis).

*HRP-2-RDTs*, histidine-rich protein 2 based rapid diagnostic tests; *Nested PCR*, nested polymerase chain reaction; *LAMP*, loop-mediated isothermal amplification; *PPV*, positive predictive value; *NPV*, negative predictive value.

For the therapeutic follow-up, HRP-2-RDTs had very poor PPV (18-21%) no matter that microscopy or nested PCR was used as a gold standard (Tables 3 and 4). Considering the poor performance of microscopy as the gold standard, both nested PCR and LAMP exhibited moderate PPV (Table 3). When nested PCR acted as a reference standard, LAMP showed excellent PPV and NPV, in terms of evaluating the efficacy of treatment (Table 4).

## Discussion

This study has shown that LAMP with high PPV and NPV as compared to nested PCR, can be applied for malaria therapeutic follow-up. HRP-2-RDTs have similar sensitivity as microscopy but less specificity, which can either induce to underdiagnosis because of the moderate NPV or over-diagnosis due to the poor PPV.

Methods for the detection of malaria are central to an elimination programme. Current diagnostic methods applied in endemic areas are either time-consuming, or insensitive for use in conditions of low infection [13,24]. Optical microscopy is often taken as the gold standard for diagnosis, but its limited sensitivity due to low parasitaemia, common in low endemic areas, makes it unreliable [25,26]. A previous study using Bayesian latent class models revealed the danger of statistical analysis based on microscopy as a reference test [15]. Using nested PCR as a gold standard, this study has shown that good agreement with nested PCR is achieved with LAMP. LAMP for molecular detection of *P. falciparum* has been compared with other diagnostic tests, but both sensitivity and specificity of LAMP varied in different studies [23,27-30]. A recent report on the evaluation of LAMP for malaria diagnosis at a field clinic found that PPV and NPV of LAMP were 100% and 98%, respectively, which are similar to our results, indicating that LAMP is an effective tool for malaria diagnosis in a field setting [29].

The inconsistent results between nested PCR and LAMP were found in two cases (one with PCR negative but LAMP positive while one with PCR positive but LAMP negative). This discrepancy may be expected when both nested PCR and LAMP are performed by two technicians independently. It has been reported that the sensitivity of a given PCR assay varies between laboratories. Although, the variations are relatively minor, they primarily diminish the ability to detect low-level and mixed infections [31]. These problems may also occur in LAMP based assays. The case with LAMP positive but nested PCR negative was falciparum malaria with extreme low parasitaemia while the case with nested PCR positive but LAMP negative harboured a mixed infection with *P. falciparum* and *P. vivax.* Low parasitaemia and mixed infection may limit the detection, even by use of molecular methods, but this cannot be the excuse for the contradiction in results between LAMP and nested PCR.

It has been well documented that blood compositions, such as haemoglobin and IgG/IgM, can interfere with the performance of PCR [32-34]. A rapid extraction of DNA from filter paper may be favourable for LAMP but a conventional method is needed for the nested PCR. In practice, the risk of variation in results increases as in the case of nested PCR where more steps in the preparation of DNA purification are required. Although errors in the performance of nested PCR and LAMP cannot be excluded, a good participation of well-trained technicians and good controls greatly reduce mistakes. To make sure no amplification in the negative controls, the three- room rule has been applied for the setup; one for DNA extraction, one for reaction preparation, and one for amplification – cycler (PCR) and heater (LAMP). The three- room policy has minimized the false positivity caused by contamination. The cause for discrepancy in results between nested PCR and LAMP is not clear and no available tests can verify the difference unless they were reconciled and repeated. The need of accurate, sensitive, affordable and easy to run for the LAMP warrants further investigation. More sensitive LAMP assay to detect malaria parasites targeted mitochondria DNA has been developed [27].

Malaria can be over-diagnosed if febrile children had positive HRP-2-RDTs but negative microscopy. One third of children (23/68) with positive HRP-2-RDTs were negative by microscopy and this can be attributed to low parasite density or to the delayed clearance of circulating HRP-2 antigen. Further verification by molecular tests, positive results from nested PCR and LAMP (10 out of 23) provided the evidence for the low parasitaemia while negative results from nested PCR and LAMP (13 out of 23) explained the possibility for the residual circulating HRP-2 antigens. Other causes for false-positive RDT results, such as gametocytaemia and the presence of the serum rheumatoid factor cannot be excluded [24,35-37]. At follow-up on day 7, these 13 children revealed the same test results as prior to the initial treatment which suggests over-treatment for malaria. Although the causes of fever origin were unknown, these children became afebrile and left without any sequelae after the anti-malaria treatment.

It has been well documented that individuals with low parasite density act as reservoirs for transmission [4,6,8] and are also more prone to having malaria attack [5,7] In this study, all febrile children with positive RDTs were treated as malaria at the first instance because patients with positive RDTs in a suspected epidemic can be highly suggestive of falciparum malaria [3]. In addition, it is not wise to delay the treatment for falciparum malaria until the nested PCR result is available, which takes several hours. LAMP is simpler and faster and can be a potential tool to replace nested PCR, but it still takes time to obtain results as the laboratory is usually distant from collection points. While the costs for the LAMP assay are only about a tenth of that for the conventional PCR [17], the reagents and enzymes are still expensive and may restrict its use in malaria-endemic areas. Future development of RDT-based LAMP for the malaria diagnosis should always take into account the affordability by the users in resource-limited countries [30]. With improvement of infra-structures of health system in the future, LAMP can be useful as a point-of-care test.

In tropical Africa, the differentiation of malaria from other causes of fever in the absence of microscopy is notoriously difficult. Even febrile children were malaria positive by microscopy it is a challenge to distinguish children who really do have severe malaria from those who have severe febrile illness but coincidental parasitaemia, who may have another infection. Although recent studies reported a strong correlation between plasma PfHRP2, disease severity, and outcome, and suggest that plasma PfHRP2 is a prognostic indicator in African children with severe falciparum malaria [38,39], measurement of plasma PfHRP2 was not available in this study. On the other hand, current HRP2-based RDTs cannot distinguish between asexual parasitaemia and gametocytaemia, which also contribute to the production of HRP2. Evidence showed that the RDT line intensity did not correlate with parasite density, days of parasitaemia, or disease severity [40]. Therefore, RDTs cannot be considered quantitative.

False-negative malaria RDT results can occur due to a prozone-like effect in high-density infections [41]. In this study, two children with high parasite density (~100,000/μl) were negative for HRP-2-RDTs but positive for microscopy, nested PCR, and LAMP. Their initial negative HRP-2-RDT became positive after a 10-dilution of blood was made. However, low parasitaemia below the detection limit of microscopy is the major cause for false-negative HRP-2-RDT results found in this study. False negativity due to the genetic heterogeneity of PfHRP-2 expression is also possible. A recent report showed that falciparum malaria parasites in Africa fail to produce HRP-2 can cause patent bloodstream infections and false-negative RDT results, which were more frequently seen in persons with asymptomatic infections [42].

Artesunate plus amodiaquine combination is one ACT recommended by the WHO for use in malaria control programmes and a first line treatment for African children with uncomplicated malaria [2]. This recommendation is now a national policy in STP. Clinical trials have shown that artesunate-amodiaquine is a highly efficacious and safe anti-malarial drug [43]. However, poor adherence cannot achieve its desired therapeutic outcome. Too many tablets is inconvenient for children and patients/guardians may discontinue the treatment when they think their children feel better after fever subsides. A new formulation of artesunate-amodiaquine (Winthrop) based on one tablet per day administration, may improve compliance.

The efficacy of the six-dose in three-day regimen of artemether/lumefantrine has been confirmed in many different patient populations around the world, cure rates on 7,14 and 28 days exceeding 95% in the evaluable population [44]. The 14-day follow-up revealed that the cure rate for second-line treatment was 100% by microscopy but 92% corrected by nested PCR and LAMP. The 28-day follow-up showed the cure rate of 100% by microscopy, but 83% corrected by nested PCR and LAMP. The lower efficacy of second-line treatment observed in this study can be attributed to the presence of drug-resistant parasites which may contribute to the spread of drug-resistant mutant strains of the malaria parasite, thus complicating the treatment scenario.

The weakness of this study was that it was not randomized and the sample size was relatively small. In addition, both nested PCR and LAMP detect the amplification of target DNA while RDTs is antigen capture and microscopy is morphological identification. With the different targets detected, comparisons among these four different tests may be limited. Latent class models have been widely used to estimate diagnostic tests performance measures, such as sensitivity and specificity, as well as the diseases prevalence, in the absence of a gold standard or perfect reference test. In spite of being frequently used as a reference technique, microscopy is in fact an imperfect gold standard, especially in low parasitaemia as in this malaria control island [15].

Although this study was carried out during the rainy season with a short period time and only older children were enrolled, malaria positive rate in febrile children was high (1/3 by microscopy or 1/2 by the nested PCR), which strongly indicates a high risk of malaria epidemics in a low transmission area, like São Tomé. After an intensive treatment and follow-up, there was no mortality observed among these febrile children.

## Conclusions

In developing countries malaria diagnosis mainly depends on microscopy or RDTs. For an elimination programme in malaria-endemic areas, however, current HRP-2-RDTs and optical microscopy are not adequate enough to evaluate the efficacy of treatment or detect low parasitaemia, especially in people with asymptomatic infection. Both sensitivity and specificity of the LAMP assay were similar to those of nested PCR. With high PPV and NPV, LAMP was the best method of the four tests for malaria diagnosis during a follow-up study. LAMP can be further developed as a point-of-care test, which is a valuable surveillance tool for guiding elimination efforts.

## Competing interests

The authors declare that they have no competing interests.

## Authors’ contributions

PWL carried out the nested PCR and conducted data analysis; DDJ assisted with the design of the study, supervised LAMP laboratory work; CTL oversaw fieldwork and collected the field data. HSR contributed to the study coordination and organized field work. VEdR helped in the study design and critically reviewed the manuscript. IFL took full responsibility for the integrity of the data and accuracy of the data and its analysis. MFS led the conceptual design, study coordination, supervision, data interpretation and drafted the manuscript. All authors read and approved the final manuscript.

## References

[B1] Lee PW Liu CT Rampao HS do Rosario VE Shaio MF Pre-elimination of malaria on the island of Príncipe Malar J 2010 9 26 10.1186/1475-2875-9-26 20089158 PMC2823607

[B2] World Health Organization World malaria report 2011 Geneva: World Health Organization

[B3] Lee PW Liu CT do Rosario VE de Sousa B Rampao HS Shaio MF Potential threat of malaria epidemics in a low transmission area, as exemplified by Sao Tome and Principe Malar J 2010 9 264 10.1186/1475-2875-9-264 20920216 PMC2955676

[B4] Von Seidlein L Drakeley C Greenwood B Walraven G Targett G Risk factors for gametocyte carriage in Gambian children Am J Trop Med Hyg 2001 65 523 527 11716108 10.4269/ajtmh.2001.65.523

[B5] Njama-Meya D Kamya MR Dorsey G Asymptomatic parasitaemia as a risk factor for symptomatic malaria in a cohort of Ugandan children Trop Med Int Health 2004 9 862 868 10.1111/j.1365-3156.2004.01277.x 15303990

[B6] Bousema JT Gouagna LC Drakeley CJ Meutstege AM Okech BA Akim IN Beier JC Githure JI Sauerwein RW Plasmodium falciparum gametocyte carriage in asymptomatic children in western Kenya Malar J 2004 3 18 10.1186/1475-2875-3-18 15202944 PMC441400

[B7] Laishram DD Sutton PL Nanda N Sharma VL Sobti RC Carlton JM Joshi H The complexities of malaria disease manifestations with a focus on asymptomatic malaria Malar J 2012 11 1 29 10.1186/1475-2875-11-1 22289302 PMC3342920

[B8] Karl S Gurarie D Zimmerman PA King CH St Pierre TG Davis TME A sub-microscopic gametocyte reservoir can sustain malaria transmission PLoS One 2011 6 e20805 10.1371/journal.pone.0020805 21695129 PMC3114851

[B9] Perkins MD Bell DR Working without a blindfold: the critical role of diagnostics in malaria control Malar J 2008 7 suppl S5 10.1186/1475-2875-7-S1-S5 19091039 PMC2604880

[B10] Bisoffi Z Gobbi F Angheben A den Ende JV The role of rapid diagnostic tests in managing malaria PLoS Med 2009 6 e1000063 10.1371/journal.pmed.1000063 19399160 PMC2667642

[B11] Milne LM Kyi MS Chiodini PL Warhurst DC Accuracy of routine laboratory diagnosis of malaria in the United Kingdom J Clin Pathol 1994 47 740 742 10.1136/jcp.47.8.740 7962629 PMC502149

[B12] Warhurst DC Williams JE Laboratory diagnosis of malaria J Clin Pathol 1996 49 533 538 10.1136/jcp.49.7.533 8813948 PMC500564

[B13] Moody A Rapid diagnostic tests for malaria parasites Clin Microbiol Rev 2002 15 66 78 10.1128/CMR.15.1.66-78.2002 11781267 PMC118060

[B14] Houze S Boly MD Le Bras J Deloron P Faucher JF PfHRP2 and PfLDH antigen detection for monitoring the efficacy of artemisinin-based combination therapy (ACT) in the treatment of uncomplicated falciparum malaria Malar J 2009 8 211 10.1186/1475-2875-8-211 19735557 PMC2754493

[B15] Goncalves L Subtil A de Oliveira R do Rosario V Lee PW Shaio MF Bayesian latent class models in malaria diagnosis PLoS One 2012 7 e40633 10.1371/journal.pone.0040633 22844405 PMC3402519

[B16] Hänscheida T Grobusch MP How useful is PCR in the diagnosis of malaria? Trends Parasitol 2002 18 395 398 10.1016/S1471-4922(02)02348-6 12377256

[B17] Poon LL Wong BW Ma EH Chan KH Chow LM Abeyewickreme W Tangpukdee N Yuen KY Guan Y Looareesuwan S Peiris JS Sensitive and inexpensive molecular test for falciparum malaria: detecting Plasmodium falciparum DNA directly from heat-treated blood by loop-mediated isothermal amplification Clin Chem 2006 52 303 306 16339303 10.1373/clinchem.2005.057901

[B18] Han ET Watanabe R Sattabongkot J Khuntirat B Sirichaisinthop J Iriko H Jin L Takeo S Tsuboi T Detection of four Plasmodium species by genus- and species-specific loop-mediated isothermal amplification for clinical diagnosis J Clin Microbiol 2007 45 2521 2528 10.1128/JCM.02117-06 17567794 PMC1951264

[B19] Lucchi NW Demas A Narayanan J Sumari D Kabanywanyi A Kachur SP Barnwell JW Udhayakumar V Real-time fluorescence loop mediated isothermal amplification for the diagnosis of malaria PLoS One 2010 5 e13733 10.1371/journal.pone.0013733 21060829 PMC2966401

[B20] Snounou G Viriyakosol S Jarra W Thaithong S Brown KN Identification of the four human malaria parasite species in field samples by the polymerase chain reaction and detection of a high prevalence of mixed infections Mol Biochem Parasitol 1993 58 283 292 10.1016/0166-6851(93)90050-8 8479452

[B21] Bereczky S Mårtensson A Gil JP Färnert A Rapid DNA extraction from archive blood spots on filter paper for genotyping of Plasmodium falciparum Am J Trop Med Hyg 2005 72 249 251 15772315

[B22] Liang SY Chan YH Hsia KT Lee JL Kuo MC Hwa KY Chan CW Chiang TY Chen JS Wu FT Ji DD Development of loop-mediated isothermal amplification assay for detection of Entamoeba histolytica J Clin Microbiol 2009 47 1892 1895 10.1128/JCM.00105-09 19321720 PMC2691116

[B23] Paris DH Imwong M Faiz AM Hasan M Yunus EB Silamut K Lee SJ Day NP Dondorp AM Loop-mediated isothermal PCR (LAMP) for the diagnosis of falciparum malaria Am J Trop Med Hyg 2007 77 972 976 17984362

[B24] Wongsrichanalai C Barcus MJ Muth S Sutamihardja A Wernsdorfer WH A review of malaria diagnostic tools: microscopy and rapid diagnostic test (RDT) Am J Trop Med Hyg 2007 77 Suppl 6 119 127 18165483

[B25] Collier JA Longmore JM The reliability of the microscopic diagnosis of malaria in the filed and in the laboratory Ann Trop Med Parasitol 1983 77 113 117 6192774 10.1080/00034983.1983.11811683

[B26] Payne D Use and limitations of light microscopy for diagnosing malaria at the primary health care level Bull World Health Organ 1988 66 621 626 2463112 PMC2491188

[B27] Polley SD Mori Y Watson J Perkins MD Gonzalez IJ Notomi T Chiodini PL Sutherland CJ Mitochondrial DNA targets increase sensitivity of malaria detection using loop-mediated isothermal amplification J Clin Microbiol 2010 48 2866 2871 10.1128/JCM.00355-10 20554824 PMC2916605

[B28] Poschl B Waneesorn J Thekisoe O Chutipongvivate S Karanis P Comparative diagnosis of malaria infections by microscopy nested PCR, and LAMP in northern Thailand Am J Trop Med Hyg 2010 83 56 60 10.4269/ajtmh.2010.09-0630 20595478 PMC2912576

[B29] Sirichaisinthop J Buates S Watanabe R Han ET Suktawonjaroenpon W Krasaesub S Takeo S Tsuboi T Sattabongkot J Evaluation of loop mediated isothermal amplification (LAMP) for malaria diagnosis in a field setting Am J Trop Med Hyg 2011 85 594 596 10.4269/ajtmh.2011.10-0676 21976556 PMC3183761

[B30] Abdul-Ghani R Al-Mekhlafi AM Karanis P Loop-mediated isothermal amplification (LAMP) for malarial parasites of humans: Would it come to clinical reality as a point-of-care test? Acta Trop 2012 122 233 240 10.1016/j.actatropica.2012.02.004 22366670

[B31] Proux S Suwanarusk R Barends M Zwang J Price RN Leimanis M Kiricharoen L Laochan N Russell B Nosten F Snounou G Considerations on the use of nucleic acid-based amplification for malaria parasite detection Malar J 2011 10 323 10.1186/1475-2875-10-323 22034851 PMC3219859

[B32] Akane A Matsubara K Nakamura H Takahashi S Kimura K Identification of the heme compound copurified with deoxyribonucleic acid (DNA) from bloodstains a major inhibitor of polymerase chain reaction (PCR) amplification J Forensic Sci 1994 39 362 372 8195750

[B33] Abu Al-Soud W Jönsson LJ Rådstr MP Identification and characterization of immunoglobulin G in blood as a major inhibitor of diagnostic PCR J Clin Microbiol 2000 38 345 350 10618113 10.1128/jcm.38.1.345-350.2000 PMC88721

[B34] Abu Al-Soud W Rådstro MP Purification and characterization of PCR-inhibitory components in blood cells J Clin Microbiol 2001 39 485 493 10.1128/JCM.39.2.485-493.2001 11158094 PMC87763

[B35] Murray CK Gasser RA Jr Magill AJ Miller RS Update on rapid diagnostic testing for malaria Clin Microbiol Rev 2008 21 97 110 10.1128/CMR.00035-07 18202438 PMC2223842

[B36] Bell D Peeling RW Evaluation of rapid diagnostic tests: malaria Nature Rev Microbiol 2006 4 suppl 34 38 10.1038/nrmicro1524 17034070

[B37] World Health Organization Methods of field trials of malaria rapid diagnostic tests 2009 Geneva: World Health Organization

[B38] Dondorp AM Desakorn V Pongtavornpinyo W Sahassananda D Silamut K Chotivanich K Newton PN Pitisuttithum P Smithyman AM White NJ Day NPJ Estimation of the total parasite biomass in acute falciparum malaria from plasma PfHRP2 PLoS Med 2005 2 e204 10.1371/journal.pmed.0020204 16104831 PMC1188247

[B39] Hendriksen ICE Mwanga-Amumpaire J von Seidlein L Mtove G White LJ Olaosebikan R Lee SJ Tshefu AK Woodrow C Amos B Karema C Saiwaew S Maitland K Gomes E Pan-Ngum W Gesase S Silamut K Reyburn H Joseph S Chotivanich K Fanello CI Day NPJ White NJ Dondorp AM Diagnosing severe falciparum malaria in parasitaemic African children: a prospective evaluation of plasma PfHRP2 measurement PLoS Med 2012 9 e1001297 10.1371/journal.pmed.1001297 22927801 PMC3424256

[B40] Bell D Wongsrichanalai C Barnwell JW Ensuring quality and access for malaria diagnosis: how can it be achieved? Nature Rev Microbiol 2006 4 suppl 7 20 10.1038/nrmicro1474 16912713

[B41] Luchavez J Baker J Alcantara S Alcantara S Belizario V Jr McCarthy JS Bell D Laboratory demonstration of a prozone-like effect in HRP2-detecting malaria rapid diagnostic tests: implications for clinical management Malar J 2011 10 286 10.1186/1475-2875-10-286 21957869 PMC3214175

[B42] Koita OA Doumbo OK Ouattara A Tall LK Konare A Diakite M Diallo M Sagara I Masinde GL Doumbo SN Dolo A Tounkara A Traore I Krogstad DJ False-negative rapid diagnostic tests for malaria and deletion of the histidine-rich repeat region of the hrp2 gene Am J Trop Med Hyg 2012 86 194 198 10.4269/ajtmh.2012.10-0665 22302847 PMC3269266

[B43] Oyakhirome S Potschke M Schwarz NG Dornemann J Laengin M Salazar CO Lell B Kun JF Kremsner PG Grobusch MP Artesunate-amodiaquine combination therapy for falciparum malaria in young Gabonese children Malar J 2007 6 29 10.1186/1475-2875-6-29 17352806 PMC1831475

[B44] Kern SE Tiono AB Makanga M Gbadoe AD Premji Z Gaye O Sagara I Ubben D Cousin M Oladiran F Sander O Ogutu B Community screening and treatment of asymptomatic carriers of Plasmodium falciparum with artemether- lumefantrine to reduce malaria disease burden: a modeling and simulation analysis Malar J 2011 10 210 10.1186/1475-2875-10-210 21801345 PMC3161019

